# Urea for treatment of acute SIADH in patients with subarachnoid hemorrhage: a single-center experience

**DOI:** 10.1186/2110-5820-2-13

**Published:** 2012-05-30

**Authors:** Charalampos Pierrakos, Fabio Silvio Taccone, Guy Decaux, Jean-Louis Vincent, Serge Brimioulle

**Affiliations:** 1 Department of Intensive Care, Erasme Hospital, Université Libre de Bruxelles, Brussels, Belgium; 2 Department of Internal Medicine, Erasme Hospital, Université Libre de Bruxelles, Brussels, Belgium; 3 Department of Intensive Care, Erasme University Hospital, Route de Lennik, 808, Brussels, B-1070, Belgium

**Keywords:** Hyponatremia, SIADH, Sodium, Subarachnoid hemorrhage, Urea

## Abstract

**Background:**

Hyponatremia occurring as a result of the syndrome of inappropriate antidiuretic hormone secretion (SIADH) or cerebral salt wasting syndrome is a common complication in patients with subarachnoid hemorrhage (SAH). The efficacy and safety of urea as treatment for SIADH-induced hyponatremia has not been reported in this population.

**Methods:**

This is a retrospective analysis of all patients admitted to our department for nontraumatic SAH between January 2003 and December 2008 (n = 368). All patients with SIADH-induced hyponatremia (plasma sodium < 135 mEq/L, urine sodium > 20 mEq/L, and osmolality > 200 mOsm/kg; absence of overt dehydration or hypovolemia; no peripheral edema or renal failure; no history of adrenal or thyroid disease) routinely received urea *per os* when hyponatremia was associated with clinical deterioration or remained less than 130 mEq/L despite saline solution administration.

**Results:**

Forty-two patients developed SIADH and were treated with urea. Urea was started after a median of 7 (IQR, 5–10) days and given orally at doses of 15–30 g tid or qid for a median of 5 (IQR, 3–7) days. The median plasma sodium increase over the first day of treatment was 3 (IQR, 1–6) mEq/L. Hyponatremia was corrected in all patients, with median times to Na^+^ >130 and >135 mEq/L of 1 (IQR, 1–2) and 3 (IQR, 2–4) days, respectively. Urea was well tolerated, and no adverse effects were reported.

**Conclusions:**

Oral urea is an effective and well-tolerated treatment for SIADH-induced hyponatremia in SAH patients.

## Background

Hyponatremia, defined as a plasma sodium concentration less than 135 mEq/L [1], is a common complication in patients with subarachnoid hemorrhage (SAH) [2,3]. Hyponatremia associated with hypertonicity, e.g., because of hyperglycemia or mannitol therapy, does not require any specific therapy in the absence of severe neurological alterations. However, when hyponatremia is associated with hypotonicity, the shift of water from the extracellular to intracellular fluid can contribute to worsening cerebral edema and intracranial hypertension, promoting seizures and further compromising neurological recovery [4]. Thus, early diagnosis and effective treatment of hyponatremia are crucial for patients with SAH. In such patients, hypotonic hyponatremia often is the result of the syndrome of inappropriate antidiuretic hormone secretion (SIADH) [3,5,6]. The main differential diagnosis in this setting is with the cerebral salt wasting syndrome (CSWS) [7,8], although the existence of this syndrome is still debated [5,8,9].

SIADH is the result of excessive secretion of antidiuretic hormone (ADH) or ADH-like substances that cause water retention. The resultant increased extracellular fluid and increased blood volume cause water and sodium diuresis, which reverse the blood volume expansion but further contribute to hyponatremia [1,10]. SIADH is, therefore, best treated by restriction of water intake to less than 500 ml/day, which can be difficult to achieve in patients with SAH for several reasons. First, many of these patients are unconscious and must be fed enterally, which results in a fluid intake of 1–2 L daily; second, hypovolemia must be avoided in patients with SAH, because it is associated with an increased risk of vasospasm and cerebral ischemia [11]. Therapeutic alternatives to water restriction include hypertonic solutions and albumin, but their efficacy is controversial [1,10]. Vasopressin receptor antagonists have been proposed [12,13], but they are expensive and can result in changes in plasma sodium that are too rapid or excessive. An easy and less expensive therapeutic option is the administration of urea [10,14]. Urea acts by inducing osmotic water elimination and by promoting passive sodium reabsorption in the ascending limb of the loop of Henle [14]. Plasma sodium and tonicity increase progressively, without causing hypertonicity. Reported adverse effects have been limited to gastrointestinal discomfort. In our department, we regularly use urea in acute SIADH occurring after acute brain injury. We recently published a retrospective review of urea use in a heterogeneous population of intensive care unit (ICU) patients [15]; however, different causes of hyponatremia and nonstandardized sodium administration were important confounding factors in the interpretation of these data. The purpose of the present study was to describe the efficacy and safety of urea for the treatment of acute SIADH in a more homogeneous population of patients with nontraumatic SAH.

## Methods

We reviewed the medical charts of 368 patients admitted to our 35-bed Department of Intensive Care for nontraumatic SAH during a 6-year period (January 2003 to December 2008). All patients who developed hyponatremia (Na <135 mEq/L) that persisted for more than 24 hours were evaluated for a diagnosis of SIADH as defined by the following standard criteria [6]: a) plasma sodium < 135 mEq /L, urine sodium > 20 mEq/L, and urine osmolality > 200 mOsm/kg; b) no overt dehydration or peripheral edema; c) no renal failure, defined as a serum creatinine ≤1.2 mg/dL; d) no previous history of adrenal insufficiency, hypothyroidism, liver cirrhosis, or heart failure [5]. We excluded patients with uncontrolled hyperglycemia (>200 mg/dL) at the time of diagnosis and patients treated with steroids, mannitol, or diuretics. The study was approved by the institutional Ethics Committee (Comité d'Ethique Hospitalo-Facultaire Erasme-ULB, reference number OM021), which waived the need for informed consent. Patients treated with urea were identified from the department database. No patients included in a previous publication [15] were included in the present cohort.

Our therapeutic protocol for SAH is standardized. All patients typically receive 3 liters of saline solution (NaCl 0.9%) daily, with additional fluids for intravenous medications and enteral nutrition, if necessary. This strategy is aimed at avoiding hypovolemia; no therapy associating induced hypertension, hypervolemia, and hemodilution (the so-called triple-H therapy) is initiated in the absence of symptomatic vasospasm. If hyponatremia develops, fluid intake is not restricted, but all intravenous fluids (including those for drug administration) are changed to isotonic saline. Hypertonic solutions or preventive hydrocortisone are not used. Plasma sodium is monitored at least every 12 hours. When SIADH is diagnosed, urea therapy is part of a standardized therapeutic protocol in our ICU and is initiated when hyponatremia is associated with clinical deterioration (defined as a reduction of at least 2 points in the Glasgow Coma Score [GCS]) or remains < 130 mEq/L despite NaCl 0.9% administration. Urea (Certa, Braine l’Alleud, Belgium) is given as 15 to 30 g doses of a 99% pure crystalline preparation, dissolved in 50 mL of water and administered *per os* or by nasogastric tube. The dose is repeated every 6 or 8 hours in most patients, and occasionally every 4 hours with a maximum of 180 g per day. Patient management, including the indication for urea and its dose, are discussed at least once daily with a senior ICU staff member. Urea therapy is discontinued after at least 48 hours of sodium levels >135 mEq/L.

Data collection included patient demographics, World Federation of Neurosurgeons Scale [16] and Fisher scale [17] scores, the location and treatment of the aneurysm, the urea doses, daily plasma and urine electrolytes and urine osmolality, daily fluid balance, ICU, and hospital lengths of stay and outcomes. Creatinine clearance (CrCL) on admission was measured from urinary creatinine excretion using the following formula: CrCL (mL/min) = [daily urine output (mL) × urinary creatinine (mg/dL)] / [urine output time collection (min) × serum creatinine (mg/dL)]. Urea treatment efficacy was evaluated as the time needed to restore safe (>130 mEq/L) and normal (>135 mEq/L) plasma sodium levels. Overcorrection was defined as an increase in plasma sodium >12 mEq/L during any 24-hour period [18]. We evaluated potential adverse effects of urea therapy, including gastrointestinal intolerance and excessive increases in blood urea. In patients with altered consciousness, gastrointestinal intolerance was defined as a gastric residual volume > 300 mL or repeated vomiting (>3/day), developing during urea therapy. Excessive increase in blood urea was defined as values > 80 mg/dL (normal range, 15–40 mg/dL). Daily serum creatinine (sCr) levels also were collected during urea therapy; renal dysfunction was defined as an increase in sCr of ≥0 .3 mg/dL compared with baseline levels. We also recorded the occurrence of sepsis and heart failure during the ICU stay, because these conditions may complicate hyponatremia. Finally, we recorded arterial lactate (normal range, <2 mEq/L) and central venous pressure (CVP) levels daily; the need for fluid and vasopressor administration for reasons other than vasospasm was considered as a possible indicator of hypovolemia. Long-term follow-up was retrospectively assessed by using the Glasgow Outcome Scale (GOS: 1 = Dead; 2 = Vegetative State; 3 = Severely Disabled; 4 = Moderately Disabled; 5 = Good Recovery) from the medical charts of the 6-month (±2 weeks) neurosurgical visit.

Statistical analyses were performed by using the SPSS 13.0 package. Discrete variables were compared by chi-square or Fisher’s exact tests. Normally distributed continuous variables were expressed as mean ± SD and compared by Student’s *t* tests. Other continuous variables were expressed as median (25th – 75th percentiles) and compared by Mann–Whitney tests. Repeated measures ANOVA for groups per time interaction and group and time comparisons, with Bonferroni correction for *post hoc* analysis, was used. Linear correlation was calculated using Spearman correlation coefficient. A *p* value < 0.05 was considered statistically significant.

## Results

Among 368 patients with SAH during the study period, 100 patients (27%) developed hyponatremia for more than 24 hours. Forty-two patients met the diagnostic criteria for SIADH and received urea. The characteristics of these patients are shown in Table 1. Almost all patients were scored grade III or IV on the Fisher scale, and 37 patients had an aneurysm explaining the SAH. At ICU admission, 41 patients had a normal sodium concentration. Hyponatremia was diagnosed after a median of 4 days (Table 2). All patients had a positive fluid balance over the 48 hours preceding urea administration (Table 3). None of these patients developed sepsis, renal, or heart failure during their ICU stay.

**Table 1:** Patient characteristics

	
Age (yr)	56 ± 12
Men / women	20 / 22
WFNS score	
I	15
II	5
III	5
IV	8
V	9
Fisher score	
I	0
II	1
III	18
IV	23
Aneurysms	
Anterior circulation	23
Posterior circulation	14
Not found	5
Endovascular intervention	33
Surgical intervention	4
Symptomatic vasospasm	8
Mechanical ventilation on admission, n (%)	30 (73)
ICU length of stay (days)	18 (12–25)
Hospital length of stay (days)	29 (21–35)
ICU mortality, n (%)	10 (23)
Hospital mortality, n (%)	11 (26)

Data are expressed as counts (percentage), median (IQR) or mean ± SD.

*WFNS* World Federation Neurosurgeon Scale, *ICU* Intensive care unit.

**Table 2:** Sodium values and urea use

	
Plasma Na at admission (mEq/L)	139 ± 3
Plasma Na at diagnosis of hyponatremia (mEq/L)	131 ± 2
Days from admission to diagnosis of hyponatremia	4 (2–5)
Plasma Na before urea (mEq/L)	127 ± 2
Days from admission to start of urea treatment	7 (5–10)
Duration of urea treatment (days)	5 (3–7)
Daily urea dose (g/day)	50 (40–60)
Change in plasma Na over first 24 h (mEq/L)	3 (1–6)
Time to plasma Na > 130 mEq/L (days) (n = 25)	1 (1–2)
Time to plasma Na > 135 mEq/L (days) (n = 42)	3 (2–4)

Data are expressed as median (IQR) or mean (±SD).

**Table 3:** Sodium and volume balance before (day −2 and day −1) and after (day 1 and day 2) the onset of urea therapy

**Variable**	**Day −2**	**Day −1**	**Day 1**	**Day 2**	**After urea**
Fluid intake (mL/24 hr)	4411 ± 1149	3958 ± 1348	3784 ± 1090	3875 ± 1370	2567 ± 1370
Urine output (mL/24 hr)	3886 ± 1842	3757 ± 1174	4288 ± 1472	4296 ± 1692^a^	1879 ± 489
Na intake (mEq/24 hr)	349 ± 181	375 ± 171	350 ± 161	346 ± 193	141 ± 69
Urine Na (mEq/24 hr)	520 ± 244	521 ± 262	467 ± 256^a,b^	491 ± 355	78 ± 36
Urine osmolality (mOsm/Kg)	515 ± 164	498 ± 125	578 ± 119	611 ± 134^b^	418 ± 96
Serum urea (mg/dL)	30 ± 9	30 ± 6	39 ± 11^a,b^	37 ± 10^a,b^	31 ± 6
Urine urea (mg/dL)	632 ± 319	699 ± 418	1653 ± 677^a,b^	1894 ± 455^a,b^	788 ± 401

“After urea” shows data on the fifth day after urea discontinuation (n = 34). Data are presented as mean ± SD. ANOVA analysis for urine output: *p* = 0.048. ANOVA analysis for urin osmolality: *p* = 0.005. ANOVA analysis for urine Na: *p* = 0.04. ANOVA analysis for serum urea: *p* < 0.001. *P* value < 0.05 vs. day −2 (^a^), day −1 (^b^), or day 1 (^c^) in *post-hoc* Bonferroni correction.

**Figure 1:**
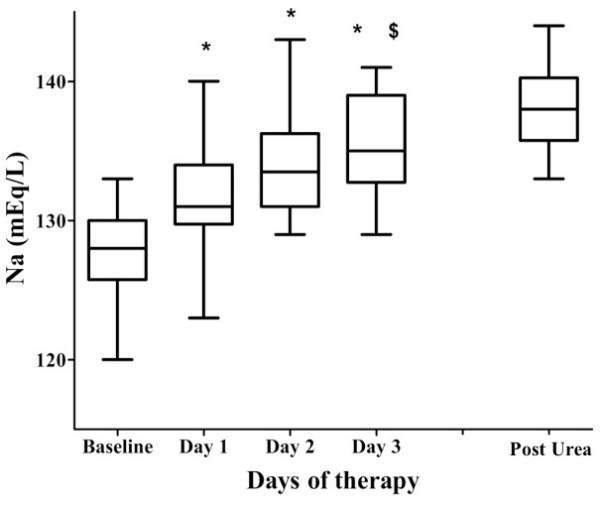
**Plasma sodium (mEq/L) at baseline (before therapy) and on days 1, 2, and 3 of urea therapy and on the fifth day after urea discontinuation (post-urea).** ANOVA analysis (excluding the last time point) for sodium changes over time – = *p* = 0.001. * = *p* < 0.01 vs. baseline, $ = *p* < 0.01 vs. day 1 in *post-hoc* Bonferroni correction.

Urea was started after a median of 7 days, given for a median of 5 days, and discontinued in all patients before ICU discharge. Urea was generally given in 15 or 30 g doses tid or qid, with a median daily dose of 50 g. Most patients (31/42) received urea through a nasogastric tube. Hyponatremia was reversed in all patients, with plasma sodium returning to greater than 130 and 135 mEq/l after median times of 1 and 3 days, respectively. Plasma sodium increased by a median of 3 (IQR, 1–6) and 5 (IQR, 3–10) mEq/L over the first and second days of treatment, respectively (Figure 1).

**Figure 2:**
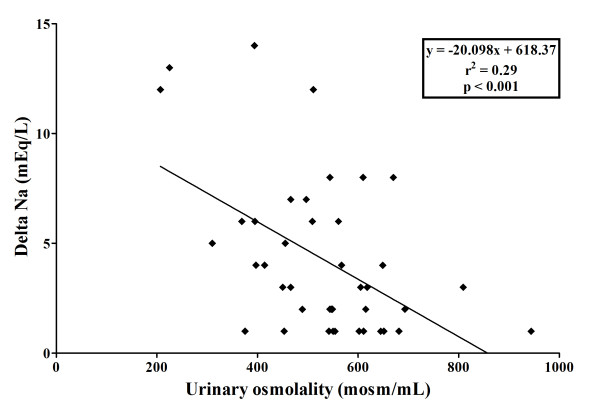
Relationship between baseline urine osmolality (mOsm/Kg) and change in plasma sodium (DeltaNa, mEq/L) during the first 24 hours of urea therapy.

Arterial lactate levels remained within normal ranges, and the CVP was > 5 mmHg in all patients; no signs of hypovolemia were noted before or during urea therapy. Compared with the day before the start of urea, urine output increased during the first day of therapy, despite a similar fluid intake (Table 3). Urine osmolality and urine urea concentrations also increased, whereas urine sodium decreased. There was an inverse correlation between baseline urine osmolality and change in plasma sodium during the first 24 hours of urea administration (Figure 2). There was no correlation between baseline plasma sodium or CrCL and change in plasma sodium.

Urea was well tolerated and no gastrointestinal, or hemodynamic adverse effects were reported. No renal dysfunction was observed during urea therapy. The plasma sodium increased ≥12 mEq/L during the first day of therapy in four patients, but no clinical deterioration was observed. Two patients developed transient hypernatremia (maximum value 149 mEq/L), which resolved within 24 hours. Sodium concentrations remained greater than 135 mEq/L in patients with sodium measurements available on the fifth day after urea discontinuation (Table 3; Figure 1), whereas urine osmolality, sodium, and urea concentrations progressively decreased. No patient had blood urea levels greater than 80 mg/dL during the treatment period.

There were no differences between ICU survivors and nonsurvivors for admission sodium levels (139 ± 3 vs. 141 ± 4 mEq/L) or in the lowest sodium levels reached during the ICU stay (127 ± 3 vs. 128 ± 2 mEq/L). The GOS at 6 months was one in 23 patients, two in 5 patients, and three in 3 patients; among the 4 patients who had overcorrection of their natremia, 3 had a GOS of 1 and 1 patient, whose GOS was 2 during hospital stay, died of massive pulmonary embolism 1 month after SAH occurrence.

## Discussion

Our results show for the first time that urea is an effective and safe treatment for hyponatremia in patients who develop SIADH and persistent hyponatremia after SAH. Most of the patients in our cohort had a poor radiological score (Fisher III or IV) and half of them had a poor neurological status (WFNS ≥ 3) on admission. Importantly, fluid and sodium administration were standardized (only NaCl 0.9% was administered and other potential therapies to reverse hyponatremia were not used), and negative fluid balance, fluid restriction, and diuretic therapy were avoided in all cases. The diagnosis of SIADH was made by using well-established criteria [6]. We did not routinely measure blood volume, but none of our patients had clinical or biological signs of hypovolemia or tissue hypoperfusion suggesting a diagnosis of CSWS.

Urea was given after saline solutions failed to prevent or reverse the hyponatremia. In these conditions of persisting hyponatremia, urea restored normal sodium levels within 3 days in half of the patients; 5 patients needed more than 5 days of therapy to restore sodium levels >135 mEq/L, but there were no urea “nonresponders” in the present series. In clinical practice, what represents significant hyponatremia is not clearly defined [1]. The lower limit of the normal range is 135 mEq/L, and SAH patients with sodium levels below this threshold have a worse outcome [19,20]; however, mild hyponatremia is rarely associated with symptoms, and treatment often is recommended only when the plasma sodium decreases below 130 mEq/L. We, therefore, defined two therapeutic endpoint values, 130 and 135 mEq/L, which were reached after a median of 1 and 3 days, respectively. The time to correction may seem longer than seen with other therapies [13], but the duration of hyponatremia before treatment was approximately 3 days, and the risks of osmotic demyelination would have been significant with quicker correction rates. Although the optimal rate of plasma sodium correction in acute hyponatremia has not been clearly defined, it is generally agreed that increases ≥12 mEq/L during 24 hours must be avoided [18,21]. In the present study, few overcorrections were observed, and none of the patients whose plasma sodium increased ≥12 mEq/L during the first day had long-term neurological impairment. Rates of overcorrection rarely have been reported in previous studies on hyponatremia therapy during SAH; only 1 of 41 patients treated with conivaptan had sodium overcorrection during therapy [12,13]. The progressive increase in plasma sodium levels, in contrast with the sharp increases sometimes reached when other therapies are used, may help to limit clinical complications. Moreover, experimental data suggest the concept that urea has a protective effect against the osmotic demyelination syndrome [22].

Urea increased renal water excretion due to osmotic diuresis and reduced urinary sodium loss. Constant fluid intake was maintained in all patients, and excess water was eliminated without noticeable arterial hypotension. This approach seems to represent a better option than fluid restriction, with potential complications of delayed cerebral infarcts in these patients at high risk of vasospasm. Importantly, there were no adverse effects. Urea is rapidly absorbed from the gastrointestinal tract [22,23] and may cause gastric discomfort [14]; however, most patients received the urea though a nasogastric tube, without adverse events. Blood urea levels increased only moderately during therapy, and this is consistent with increased urinary urea excretion, ultimately resulting in effective osmotic water excretion.

This study was not designed to identify the optimal strategy or most appropriate dose of urea therapy. However, the response to urea was not correlated with baseline plasma sodium, suggesting that urea is effective even in mild hyponatremia. In contrast, there was an inverse correlation between baseline urine osmolality and the rate of plasma sodium correction, suggesting that the higher the urine osmolality, the lower the ability of urea at the doses used to induce a rapid osmotic diuresis and to reverse hyponatremia. A similar finding has been reported in other studies and has been attributed to a reduced ability to excrete the excessive water when urine osmolality is high [24].

Alternative therapeutic options for preventing or reversing hyponatremia in neurocritical patients include albumin, fludrocortisone, hypertonic saline, and vasopressin receptor antagonists, such as conivaptan. The effects of albumin in limiting natriuresis have been reported in only one study [25], and they remain controversial [26]. Fludrocortisone enhances sodium retention through its mineralocorticoid properties, but its ability to correct hyponatremia is limited and it contributes to fluid overload [27,28]. Hypertonic saline solutions can increase plasma sodium concentration efficiently and very rapidly, but they also increase blood volume and the risk of pulmonary edema and heart failure as well as of neurological complications. Their effects are generally transient because the stimuli for water retention and secondary natriuresis remain present [29]. Vasopressin receptor antagonists represent a promising option, and conivaptan has been studied in two series of neurointensive care patients. In one study, a single dose of 20 mg of conivaptan increased plasma sodium by at least 4 mEq/L in 13 of 19 hyponatremic patients and maintained the sodium improvement for 3 days in most of the patients [12]. In another study, conivaptan increased plasma sodium by at least 6 mEq/L in 19 of 22 euvolemic hyponatremic patients and maintained its effects for an average of 13 hours [13]. However, the studies included only 12 patients with SAH, and fludrocortisone or hypertonic saline also were given in some patients. Moreover, conivaptan often was limited to a single bolus, and a third of the patients became hyponatremic when conivaptan was discontinued. Practical limitations to vasopressin receptor antagonists include the costs of the drug and the unpredictability of the response amplitude and duration [1]. Diuretics can be used to induce osmotic diuresis; however, they are likely to induce hypovolemia in this setting, with an increased risk of delayed cerebral ischemia [10]. Finally, demeclocycline can reverse hyponatremia induced by SIADH, but several adverse events, including nephrotoxicity, have been reported [30].

Our study has some limitations. First, the retrospective nature of the study may have limited the collection of pertinent clinical or biological data, such as adverse events. Second, we did not specifically record the neurological status or intracranial pressure according to plasma sodium values, so that the relationship between sodium changes and clinical changes cannot be evaluated. Also, we did not measure plasma osmolality. Third, no specific protocol was used for urea administration, and regimen changes were at the physician’s discretion, therefore, the optimal dose of urea and its impact on sodium level changes cannot be determined. Fourth, we specifically studied SAH patients, and our findings can be extended to other patients only with caution. Fifth, the purpose of this study was not to evaluate all the different pathophysiological mechanisms that underlie hyponatremia after acute brain injury, and no specific conclusions can be made about the effects of urea in other conditions associated with sodium imbalance in this setting. Sixth, we consider the serum urea levels reached (<80 mg/dL) to be safe, because most of the complications related to uremia occur with urea concentrations >200 mg/dL; however, we cannot exclude that complications may arise at lower urea levels. Finally, the efficacy of urea compared with other available treatments needs to be determined in large, prospective studies.

## Conclusions

Urea is an effective treatment for SIADH-related hyponatremia in patients with SAH. It is generally well-tolerated and can help to avoid fluid restriction. Dose titration and plasma sodium response rate should be further studied prospectively. Also, the efficacy of urea therapy compared with other drugs, such as albumin, hydrocortisone, hypertonic saline solution, and vasopressin receptor antagonists, remains to be demonstrated.

## Abbreviations

ANOVA: analysis of variance; CSWS: cerebral salt wasting syndrome; CVP: cerebral venous pressure; ICU: intensive care unit; SAH: subarachnoid hemorrhage; SIADH: syndrome of inappropriate antidiuretic hormone secretion; WFNS: World Federation of Neurosurgeons scale.

## Competing interests

The authors declare that they have no competing interests.

## Authors’ contributions

CP, FST, and SB conceived the study protocol; FST and SB supervised data collection; FST, SB, and GD participated in data interpretation; FST, JLV, and SB were responsible for urea management in SAH patients; CP and FST performed the literature research; CP, FST, and SB drafted the present manuscript; GD and JLV revised the manuscript. All authors read and approved the final version of the manuscript.

## References

[B1] Elhassan EA Schrier RW Hyponatremia: diagnosis, complications, and management including V2 receptor antagonists Curr Opin Nephrol Hypertens 2011 20 161 168 10.1097/MNH.0b013e3283436f14 21252664

[B2] Qureshi AI Suri MF Sung GY Straw RN Yahia AM Saad M Guterman LR Hopkins LN Prognostic significance of hypernatremia and hyponatremia among patients with aneurysmal subarachnoid hemorrhage Neurosurgery 2002 50 749 755 10.1097/00006123-200204000-00012 11904025

[B3] Rabinstein AA Bruder N Management of hyponatremia and volume contraction Neurocrit Care 2011 15 354 360 10.1007/s12028-011-9585-9 21748503

[B4] Mount DB The brain in hyponatremia: both culprit and victim Semin Nephrol 2009 29 196 215 10.1016/j.semnephrol.2009.03.021 19523569

[B5] Brimioulle S Orellana-Jimenez C Aminian A Vincent JL Hyponatremia in neurological patients: cerebral salt wasting versus inappropriate antidiuretic hormone secretion Intensive Care Med 2008 34 125 131 10.1007/s00134-007-0905-7 17952405

[B6] Ellison DH Berl T Clinical practice. The syndrome of inappropriate antidiuresis N Engl J Med 2007 356 2064 2072 10.1056/NEJMcp066837 17507705

[B7] Kao L Al-Lawati Z Vavao J Steinberg GK Katznelson L Prevalence and clinical demographics of cerebral salt wasting in patients with aneurysmal subarachnoid hemorrhage Pituitary 2009 12 347 351 10.1007/s11102-009-0188-9 19462244

[B8] Audibert G Steinmann G de Talancé N Laurens MH Dao P Baumann A Longrois D Mertes PM Endocrine response after severe subarachnoid hemorrhage related to sodium and blood volume regulation Anesth Analg 2009 108 1922 1928 10.1213/ane.0b013e31819a85ae 19448223

[B9] Oh MS Carroll HJ Cerebral salt-wasting syndrome. We need better proof of its existence Nephron 1999 82 110 114 10.1159/000045385 10364701

[B10] Decaux G Musch W Soupart A Management of hypotonic hyponatremia Acta Clin Belg 2010 65 437 445 21268962 10.1179/acb.2010.65.6.013

[B11] Bederson JB Connolly ES Jr Batjer HH Dacey RG Dion JE Diringer MN Duldner JE Jr Harbaugh RE Patel AB Rosenwasser RH Guidelines for the management of aneurysmal subarachnoid hemorrhage: a statement for healthcare professionals from a special writing group of the Stroke Council, American Heart Association Stroke 2009 40 994 1025 10.1161/STROKEAHA.108.191395 19164800

[B12] Murphy T Dhar R Diringer M Conivaptan bolus dosing for the correction of hyponatremia in the neurointensive care unit Neurocrit Care 2009 11 14 19 10.1007/s12028-008-9179-3 19123060 PMC2820273

[B13] Wright WL Asbury WH Gilmore JL Samuels OB Conivaptan for hyponatremia in the neurocritical care unit Neurocrit Care 2009 11 6 13 10.1007/s12028-008-9152-1 19003543

[B14] Decaux G Unger J Brimioulle S Mockel J Hyponatremia in the syndrome of inappropriate secretion of antidiuretic hormone. Rapid correction with urea, sodium chloride, and water restriction therapy JAMA 1982 247 471 474 10.1001/jama.1982.03320290017021 7054549

[B15] Decaux G Andres C Gankam KF Soupart A Treatment of euvolemic hyponatremia in the intensive care unit by urea Crit Care 2010 14 R184 10.1186/cc9292 20946646 PMC3219290

[B16] World Federation of Neurological Surgeons Committee Report on a Universal Subarachnoid Hemorrhage Grading Scale J Neurosurg 1988 68 985 986 3131498 10.3171/jns.1988.68.6.0985

[B17] Fisher CM Kistler JP Davis JM Relation of cerebral vasospasm to subarachnoid hemorrhage visualized by computerized tomographic scanning Neurosurgery 1980 6 1 9 10.1227/00006123-198001000-00001 7354892

[B18] Samuels MA Seifter JL Encephalopathies caused by electrolyte disorders Semin Neurol 2011 31 135 138 10.1055/s-0031-1277983 21590618

[B19] Hasan D Wijdicks EF Vermeulen M Hyponatremia is associated with cerebral ischemia in patients with aneurysmal subarachnoid hemorrhage Ann Neurol 1990 27 106 108 10.1002/ana.410270118 2301918

[B20] Wijdicks EF Vermeulen M Hijdra A van Gijn J Hyponatremia and cerebral infarction in patients with ruptured intracranial aneurysms: is fluid restriction harmful? Ann Neurol 1985 17 137 140 10.1002/ana.410170206 3977297

[B21] Sterns RH Riggs JE Schochet SS Jr Osmotic demyelination syndrome following correction of hyponatremia N Engl J Med 1986 314 1535 1542 10.1056/NEJM198606123142402 3713747

[B22] Soupart A Schroeder B Decaux G Treatment of hyponatraemia by urea decreases risks of brain complications in rats. Brain osmolyte contents analysis Nephrol Dial Transplant 2007 22 1856 1863 10.1093/ndt/gfm138 17395650

[B23] Aoyagi T Engstrom GW Evans WB Summerskill WH Gastrointestinal urease in man. I. Activity of mucosal urease Gut 1966 7 631 635 10.1136/gut.7.6.631 5957514 PMC1552633

[B24] Decaux G Musch W Clinical laboratory evaluation of the syndrome of inappropriate secretion of antidiuretic hormone Clin J Am Soc Nephrol 2008 3 1175 1184 10.2215/CJN.04431007 18434618

[B25] Mayer SA Solomon RA Fink ME Lennihan L Stern L Beckford A Thomas CE Klebanoff LM Effect of 5% albumin solution on sodium balance and blood volume after subarachnoid hemorrhage Neurosurgery 1998 42 759 767 10.1097/00006123-199804000-00048 9574640

[B26] Suarez JI Shannon L Zaidat OO Suri MF Singh G Lynch G Selman WR Effect of human albumin administration on clinical outcome and hospital cost in patients with subarachnoid hemorrhage J Neurosurg 2004 100 585 590 10.3171/jns.2004.100.4.0585 15070109

[B27] Hasan D Lindsay KW Wijdicks EF Murray GD Brouwers PJ Bakker WH van Gijn J Vermeulen M Effect of fludrocortisone acetate in patients with subarachnoid hemorrhage Stroke 1989 20 1156 1161 10.1161/01.STR.20.9.1156 2672426

[B28] Mori T Katayama Y Kawamata T Hirayama T Improved efficiency of hypervolemic therapy with inhibition of natriuresis by fludrocortisone in patients with aneurysmal subarachnoid hemorrhage J Neurosurg 1999 91 947 952 10.3171/jns.1999.91.6.0947 10584839

[B29] Hantman D Rossier B Zohlman R Schrier R Rapid correction of hyponatremia in the syndrome of inappropriate secretion of antidiuretic hormone. An alternative treatment to hypertonic saline Ann Intern Med 1973 78 870 875 4197370 10.7326/0003-4819-78-6-870

[B30] Curtis NJ van Heyningen C Turner JJ Irreversible nephrotoxicity from demeclocycline in the treatment of hyponatremia Age Ageing 2002 31 151 152 10.1093/ageing/31.2.151 11937482

